# Rapamycin rescues APC-mutated colon organoid differentiation

**DOI:** 10.1038/s41417-025-00935-3

**Published:** 2025-07-23

**Authors:** Aline Habib, Rose Mamistvalov, Dalit Ben-Yosef

**Affiliations:** 1 Institution of Reproduction and IVF, Lis Maternity Hospital, Tel-Aviv Sourasky Medical Center, Tel-Aviv, Israel; 2 CORAL - Center of Regeneration and Longevity, Tel-Aviv Sourasky Medical Center, Tel-Aviv, Israel; 3 Department of Cell and Developmental Biology, Sackler Faculty of Medicine, Sagol School of Neuroscience, Tel-Aviv University, Tel-Aviv, Israel

**Keywords:** Cancer stem cells, Colorectal cancer, Cancer genetics

## Abstract

Familial adenomatous polyposis (FAP) is an autosomal dominant disorder characterized by germline mutations in the adenomatous polyposis coli (APC) gene. This leads to numerous colorectal adenomas and a high risk of colorectal cancer (CRC). Our stem cell-derived colon organoid model revealed that a heterozygous APC mutation is sufficient to induce colorectal cancer formation. We found a link between APC mutation type, organoid maturation and FAP severity. Here, we show that severe germline mutations in hESCs employ diverse mechanisms of carcinogenesis. FAP1-hESCs expressing a truncated 332-amino acid protein exhibited a hyperactivated mTOR pathway, including PTEN inactivation and increased S6K1 and eIF4E activation. This affected oncogenic c-Myc expression and contributed to apoptosis resistance. Rapamycin treatment restored differentiation potential in FAP1 organoids but not FAP2 organoids, which expressed a larger truncated protein without mTOR pathway activation. Our in vitro colon organoids system findings were validated in human patients. Notably, a colon from a FAP1 patient exhibited high expression of mTOR pathway proteins. These findings highlight the potential of rapamycin for personalized therapy in FAP patients with distinct mTOR-mediated APC mutations. Our colon organoid model is valuable for studying CRC and developing new diagnostic, preventive, and therapeutic approaches to prevent or delay tumorigenesis in FAP patients.

## Scientific background

Colorectal cancer (CRC) is a leading cause of cancer-related mortality globally and ranks as the third most prevalent cancer [1]. It develops through a multi-step process characterized by the accumulation of genetic and epigenetic alterations that transform healthy colon and rectal epithelial cells into malignant tumors. This progression typically involves the sequential transformation from normal tissue to adenomatous polyps and eventually invasive carcinoma [2–4]. Loss of APC function due to germline mutations is an early and critical event in the adenoma-carcinoma sequence [2, 5, 6]. Familial adenomatous polyposis (FAP) is an autosomal dominant hereditary disorder that significantly increases the risk for CRC. It is caused by a germline mutation in the adenomatous polyposis coli (APC) gene, a critical tumor suppressor gene [5, 7–9]. Individuals with FAP usually develop hundreds to thousands of colorectal adenomas during adolescence, with a nearly 100% lifetime risk of progression to CRC unless prophylactic colectomy is performed [10–13]. The APC gene, located on chromosome 5q21-q22, encodes a large multi-domain protein that negatively regulates Wnt signaling by controlling cellular levels of β-catenin, which is crucial for cell proliferation, differentiation, and apoptosis [6, 14].

Different types of cancers upregulate the mammalian target of rapamycin (mTOR) signaling which can contribute to tumor development and progression by increasing cell proliferation and survival [15–17]. The hyperactivation of the mTOR pathway is a hallmark of CRC and a key tumorigenesis driver [18]. This dysregulation can result from mutations in the PIK3CA gene, loss of the tumor suppressor PTEN, or activation of upstream growth factor receptors promoting uncontrolled cell proliferation, survival, and metabolic reprogramming [19, 20]. Therefore, mTOR inhibitors like rapamycin offer a potential therapeutic approach for CRC [17–20].

Cells derived from FAP patients can serve as a good human model to explore the genes and pathways involved in the initiation of tumorigenic transformation in CRC in general and FAP patients in particular [21]. This study employed two previously established human embryonic stem cell (hESC) lines, derived from FAP-affected embryos, carrying distinct germline mutations in the APC gene. The FAP1-hESC line has a stop codon in amino acid 332 (exon 9) of the APC gene. While in the FAP2-hESC line, the mutation is in the splice site of intron 14, introducing a premature termination codon [22, 23]. To model the early molecular events of colon cancer development, we differentiated these FAP-hESC lines into colon organoid models mimicking the human colon’s physiological milieu, in vitro. Our previous work demonstrated that FAP-hESC lines exhibit limited differentiation capacity into colon organoids, forming simple cyst-like structures compared to control hESCs with the normal APC gene, which generate complex, molecularly mature 3D colonic structures. These findings align with the more severe clinical manifestation of FAP1/2 patients, characterized by increased polyp genotype/phenotype correlation between colon organoid maturation potential and FAP severity in the carrier parents [24–26].

Recently it was shown that rapamycin can delay and even prevent the development of large intestine cancer and extend life expectancy in Apc ^Min/+^ mice model for FAP disease [27]. Given these findings, we sought to determine if rapamycin could reverse cancer-like features in our human stem cell-based in vitro model, translating the potential therapeutic effect to a human context. Our results show that FAP1 mutation activates the mTOR pathway and the application of rapamycin effectively restored their complex organoid-forming capacity. In contrast, the FAP2 mutation did not elicit hyperactivation of the mTOR pathway, and subsequent rapamycin treatment failed to enhance the organoid differentiation. These findings highlight the potential for a personalized therapeutic approach using rapamycin in FAP patients with distinct mTOR-mediated APC mutations. Our colonic model facilitates the investigation of APC mutations in CRC initiation and enables the identification of potential therapeutic strategies to prevent or delay tumorigenesis in FAP patients.

## Material and methods

### Ethics approvals

The use of spare in-vitro fertilization (IVF) embryos following preimplantation genetic diagnosis (PGD) for the derivation of hESC lines, and the study of genetic disease was approved by the Israeli National Ethics Committee (7/04-043) and was conducted under the guidelines of the Bioethics Advisory Committee of the Israel Academy of Sciences and Humanities. Clinical data analysis of pathology samples derived during colonoscopy of FAP patients was performed under IRB (TLV-0309-23), following the signing of an informed consent.

### hESC lines

FAP-hESC lines carrying germline mutations at different locations of the APC gene were previously derived in our lab following IVF-PGD treatment for patients’ carriers of FAP, were examined in this study: Lis25_FAP1 (FAP1) and Lis30_FAP2 (FAP2) (both are females) [22, 23, 28]. hESCs were cultured on wells coated with Geltrex (A1413202, ThermoFisher), in mTeSR1 medium (85850, Stem Cell Technologies) supplemented with 100ug/ml Primocin (InvivoGen) at 37 °C with 5% CO2. hESCs were passaged at ~90% confluence using Accutase (SCR005, Stem Cell Technologies), and frozen in NutriFreez D10 (05-713-1B, Biological Industries). After passaging or thawing, 10 µM ROCK inhibitor Y-27632 (10005583, Cayman) was supplemented to mTeSR for the first 24 h to inhibit apoptosis.

### Generation of human colon organoids

Differentiation into colon organoids was induced as we described previously [28], with slight modifications. In summary, to generate a definitive endoderm, hESCs were treated with 3 μM CHIR99021 (72054, Stem Cell Technologies) and 100 ng/ml activin A (120-14E, Peprotech) in RPMI (Cellgro) medium supplemented with 2-mM GlutaMAX (Gibco) and 1X Pen/Strep (BioLab), for one day. For the second day, cells were treated with 100 ng/ml activin A in RPMI supplemented with 2% FBS (S-FBSP-EU, SERANA), 2 mM GlutaMAX and 100 U/ml Pen/Strep for three days and 20% FBS on the third day. These cells were then subjected to hindgut differentiation by treatment with 3 μM CHIR99021 and 500 ng/ml FGF4 (Peprotech) in RPMI supplemented with 1X B27 (Thermo Scientific), GlutaMAX and 100 U/ml Pen/Strep for four days. From day 8, cells were cultured in a colonic medium comprised of advanced DMEM F12 (Thermo Scientific) supplemented with 1X B27 (Gibco), 2-mM GlutaMAX, 100 U/ml Pen/Strep, 3 µM CHIR, 300 nM LDN (Peprotech) and 100 ng/ml EGF (Peprotech). This medium was used until the end of the differentiation and refreshed every two days. On day 20, the cells were disaggregated to a single cell suspension using Accutase for 10 min at 37 °C and then re-suspended in Matrigel (356231, Corning). The Matrigel beads are made by using 70 μl of Matrigel to form a solid drop-like structure, in 6 wells of Nunclon Delta Surface plates (Thermo Scientific). 10 µM of ROCK inhibitor was added for the first two days. Organoids were passed every 10 days with a 1:3 ratio.

In addition to the differentiation factors, differentiated cells at day 20 were treated with 4 ng/ml of rapamycin (53123-88-9, Fermentek)

### Western blotting analysis (WB)

Cells or colon organoids were collected and lysed in ice-cold RIPA lysis buffer (R0278, Sigma-Aldrich) containing 1-mM phenylmethylsulfonyl fluoride (PMSF) (8553, Cell Signaling, 1:1000) and phosphatase proteinase inhibitors (5872, Cell Signaling, 1:1000). 25-100 μg of the lysates were run on 4-20% SDS-polyacrylamide gel electrophoresis (SDS–PAGE) (XP04200BOX, Thermo Scientific) and then transferred to nitrocellulose (PB7320, Invitrogen) for APC protein using the Power Blotter Station (Invitrogen). The membranes were blocked with 5% BSA in 1 × PBST (0.1% Tween in X1 PBS), incubated overnight at 4 °C with primary antibody anti-p-S6K1 (CST-5364, Cell Signaling, 1:1000), anti-p-eIF4E (ab76256, Abcam, 1:5000), anti-S6K1 (CST-2217, Cell Signaling, 1:1000), anti-eIF4E (ab33766, Abcam, 1:1000) and anti-β-actin (ab8226, Abcam, 1:5000). Blots were then washed with PBST and incubated for 1 h at RT with the secondary antibody HRP anti-rabbit (CST-7074, Cell Signaling, 1:1000) or anti-mouse (CST-7076, Cell Signaling, 1:5000). The blot was developed by chemiluminescence using the Clarity Western ECL substrate (170-5060, BioRad) for 1 min then exposed to X-ray film (Azure280, Azure Biosystem).

### Immunofluorescence (IF) and Immunohistochemistry (IHC)

For hESCs staining, cells were grown in 24-well plates and fixed with 4% paraformaldehyde (PFA) (P6148, Sigma-Aldrich). Blocking was performed with a blocking solution including 5% BSA or 5% Goat serum (GS) with 0.2% Triton X 100 in PBS for permeabilization of the cells to allow detection of intracellular proteins. Cells were then incubated with primary antibody diluted in 2.5% BSA or GS blocking solution overnight at 4 °C; antibody anti-p-S6K1 (CST-5364, Cell Signaling, 1:800), anti-p-eIF4E (ab76256, Abcam, 1:500), and anti-cMyc (ab32072, Abcam, 1:75). The Next day, cells were washed three times and then incubated with secondary antibodies for 1 h at RT; anti-Rabbit IgG (A10040,1:400) and anti-Mouse IgG (A21202, 1:700), counterstained with DAPI for nucleus localization, and imaged. Bright field, phase and fluorescence images of cells were captured using an Olympus IX51 inverted light microscope.

For whole organoid staining, 40ul of Matrigel containing fully developed organoids in 8-well chamber slides (C8-1.5H-N, Cellvis) was grown for at least two days. Whole organoids were fixed in 4% PFA for 30 min at RT, washed with IF buffer: 0.2% triton and 0.05% tween in PBS. For permeabilization, organoids were incubated for 20 min in 0.5% triton in PBS, then washed with IF buffer and blocked for 30 min with blocking solution including 1% BSA. Freshly prepared primary antibodies in blocking solution were added to the fixed organoids and incubated overnight in a humidified chamber at 4°C. The organoids were washed twice with IF solution, incubated with the specific secondary antibodies for an hour at RT, and protected from light. The stained organoids were then washed and mounted using a Fluoromount Mounting Medium (00-4958-02, Thermo Scientific). 2D differentiated cells on day 20 were dissociated and plated on Geltrex-coated 8-well chamber slides. Rapamycin was added from day 20 to day 30 then cells were analyzed for IF analysis as described above. Confocal microscopy images of organoids and differentiated cell samples were obtained using a Zeiss LSM700 confocal microscope.Organoids were fixed in 4% PFA and stained for the colon markers CDX2 (235R-16, Cell Marque, 1:100), Keratin 20 (320M-16, Cell Marque, 1:100), and MUC2 (PA0155, BOND, 1:100), the stromal marker Vimentin (Dako, M0725, 1:100), KI67 (275R-16, Cell Marque, 1:200) a nuclear marker indicative of cellular proliferation, cMyc (395R-16, Cell Marque, 1:50) as an oncogenic marker and PTEN (31-1147-00, RevMab, 1:500) as mTOR pathway inhibitor. Staining was performed in the pathology lab at Tel-Aviv Sourasky Medical Center. The analysis was done by random 3 photos from each sample using light microscopy from at least two repeats.

The IHC of mTOR proteins (p-mTOR (ab109268, Abcam, 1:100), p-S6k1(#5364, Cell Signaling, 1:700) and p-eIF4E (ab76256, Abcam, 1:200) was performed in our lab on embedded samples. Slides were heated by microwave in X1 citrate unmasking solution (14746, Cell Signaling). After boiling, the heating continued at lower power for an additional 2 min. The slides were then cooled before washing with 0.05% Tween 20 in PBS (PBST) three times. The sections were then immersed in 3% H_2_O_2_ for 10 min to inactivate endogenous peroxidase and subsequently rinsed with water and 0.05% PBST for 5 min. The blocking process was performed by incubating the slides with 5% GS in PBST for 1 h at room temperature following incubation with primary antibodies overnight at 4 °C. Antibodies were diluted in SignalStain Antibody Diluent (8112, Cell Signaling). The samples were washed three times with PBST followed by 30 min of incubation with Boost Detection Reagent (HRP, Rabbit #8114 or Mouse #8125, Cell Signaling). The samples were then washed in water and PBS before incubation with the DAB Immunohistochemistry Visualization System (#8059) for 3–5 min. The reaction was terminated by submerging in water; counterstaining was performed for 3–5 min using hematoxylin (#14166, Cell Signaling) and then washed with water for 5 min. For the section dehydrated, the slides were immersed in 95% ethanol for 10 s, repeated with 100% ethanol, cleared in xylene, and mounted using immunomount.

### Annexin-V assay for apoptosis

An Annexin V-FITC MEBCYTO Apoptosis kit (4700, MBL) was used to detect apoptotic cells in colon-differentiated cells according to the manufacturer’s instructions. Differentiated cells at day 20 were dissociated using Accutase for 10 min. Cells were collected by centrifugation at 500 × *g* for 5 min at 4 °C. The cells were resuspended in 85 μL of binding buffer; next 10 μL of Annexin V-FITC and 5 μL of propidium iodide (PI) were added, and the cells were incubated at room temperature (20–25 °C) for 15 min in the dark. Then, 400 μL of binding buffer was added, and the cells were analyzed by FACSCanto flow cytometer (BD Biosciences). Annexin V-FITC-positive and PI-negative cells were considered apoptotic cells.

### Statistical analysis

For all experiments, at least two independent experiments were carried out unless otherwise stated. *p* values were calculated using the unpaired two-tailed Student’s t-test and ANOVA, both computed with SPSS, and are represented as *p < 0.05, **p < 0.01, ***p < 0.001, and ****p < 0.0001. All data are presented as the mean ± standard error.

## Results

**Fig. 1:**
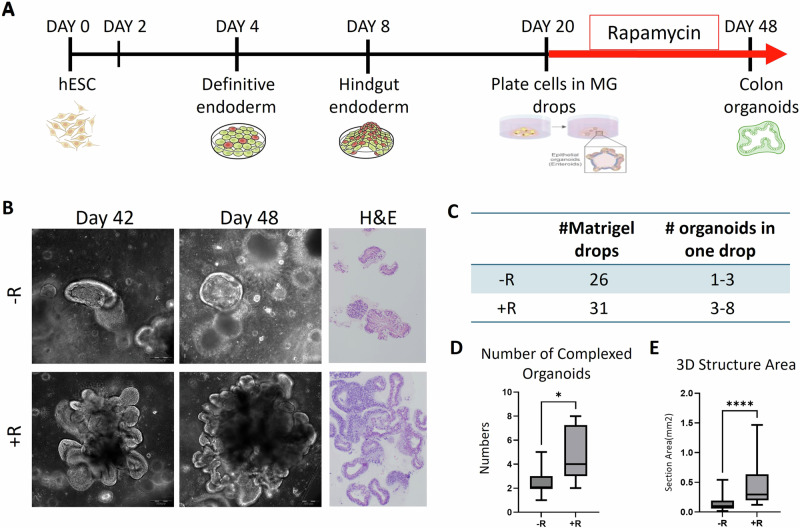
The mTOR inhibitor rapamycin improves the differentiation of FAP1-hESCs into colon organoids. **A** A schematic diagram of the colon differentiation protocol from hESCs, with the indicated timing of rapamycin treatment. **B** Representative images of 42-48 days 3D colon organoids derived from heterozygous mutated APC FAP1-hESCs cultured with and without rapamycin treatment. H&E staining presents the complexity of the organoids following rapamycin treatment**. C**, **D** The number of complex colon organoids developed by day 48 of differentiation of FAP1-hESCs cultured with and without rapamycin treatment. A total of 14 biological experiments were performed and 26–31 Matrigel drops were analyzed. Graphs present the average of complexed organoids per one Matrigel drops, values are presented as Avg ± Std. *P < 0.05, ****P < 0.001; *t* test. **E** Organoids area size of a representative FAP1-colon organoids section measured using the image processing program ImageJ, of 38 untreated and 35 rapamycin-treated organoids. Values are presented as Avg ± Std; ****P < 0.0001 (*t* test).

Expanding on our FAP-hESC colon organoid model and Rapamycin’s success in preventing APC mutant mouse cancer [27, 28], we investigate its potential to reverse cancer-like features in our in vitro human model. Initially, we performed time and dose optimization experiments for the FAP1 cells demonstrating that 4 ng/mL rapamycin treatment successfully restored the ability of FAP1-hESCs to form complex colon organoids (Fig. 1A, B). Complex organoids are self-organized three-dimensional colonic structures exhibiting crypt-like structures resembling the in vivo intestinal epithelium and expressing the colon intestinal markers CDX2 and CK20. A total of 14 experiments (26-31 Matrigel droplets) were performed in which FAP1 underwent differentiation with/without Rapamycin. Our findings revealed a significant increase in the generation of complex organoids upon rapamycin treatment from an average of 1-3 organoids per Matrigel droplet without rapamycin to 3-8 organoids /Matrigel droplet following treatment (Fig. 1C, D). Cells that were unable to undergo the direct differentiation processes for complex colon organoid formation instead developed into simple cyst-like structures. Thus, the formation of cysts actually reflects the inability of the cells to form organoids. We therefore decided to focus on organoid formation which is the true sign of the ability of the cells to model the natural differentiation into colon tissue. Rapamycin’s positive effect on FAP1 cells was further supported by the enhanced organoid complexity evident in H&E staining and by the significant increase in the 3D structural area of the organoids (Fig. 1B, E). Collectively, these results demonstrate that rapamycin can reverse the differentiation defect in FAP1-hESCs, enabling them to form mature complex colon organoids.

**Fig. 2:**
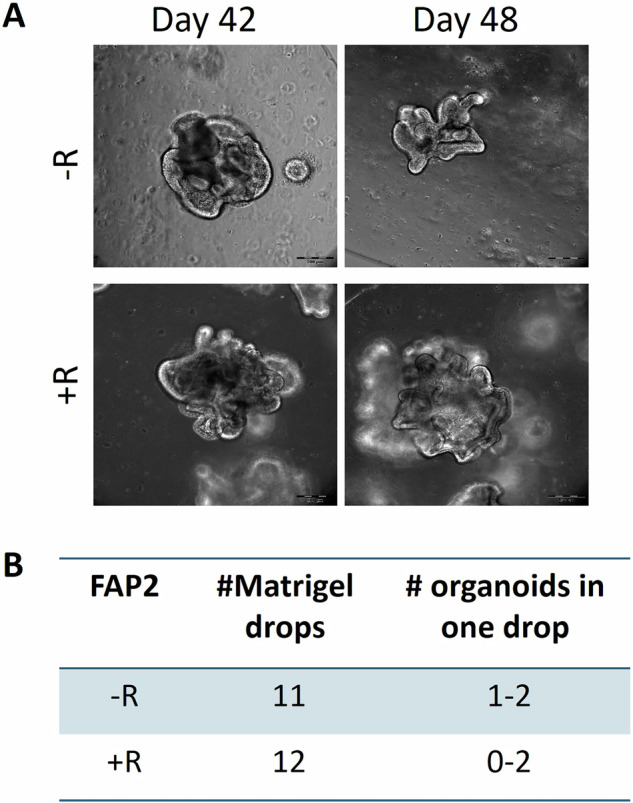
FAP2-hESC differentiation was unaffected by rapamycin. **A** Representative images of 42-48 days 3D colon organoids derived from heterozygous mutated APC FAP2-hESCs with and without rapamycin treatment. **B** The number of complex colon organoids developed by day 48 of differentiation of FAP2-hESCs with and without rapamycin treatment. Eight biological experiments were performed and 11–12 Matrigel drops were examined.

Unlike FAP1 cells, rapamycin treatment failed to improve the differentiation capacity of FAP2 cells harboring a different APC heterozygous mutation. FAP2-derived cultures remained restricted to cyst-like structures even in the presence of rapamycin (Fig. 2A). A total of 8 experiments (11–12 Matrigel droplets) were performed in which FAP2 underwent differentiation with/without Rapamycin. Nevertheless, in all experiments, rapamycin was unable to rescue organoid formation (Fig. 2B).

**Fig. 3:**
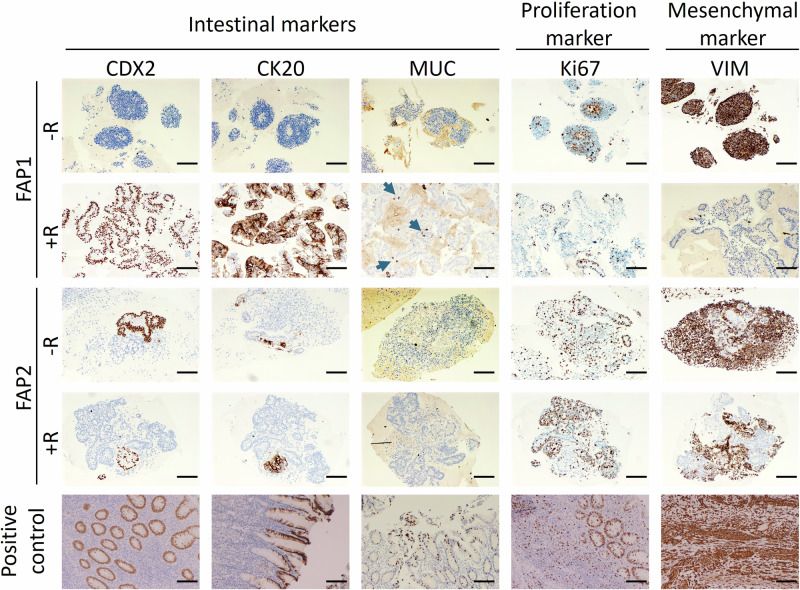
Lineage characterization of cells comprising colon organoids treated with rapamycin. Representative images of immunohistochemical staining of the intestinal epithelial markers CDX2, Keratin20 (CK20), Muc2, the proliferation marker Ki67, and the mesenchymal marker VIM, for FAP1 and FAP2 colon organoids at day 45 following rapamycin treatment (R). Non-treated cells served as a negative control, and normal colon tissue served as a positive control. Arrows point for Muc2 staining. Scale bars 200 µM. VIM vimentin, R Rapamycin.

The effect of rapamycin on organoid formation was further analyzed at the cellular level, by comparing the expression levels of different colonic markers by IHC staining. The results indicate that FAP1-treated organoids exhibit elevated expression levels of the intestinal epithelial markers (CDX2, Keratin20 (CK20), Muc2), compared to untreated FAP1 organoids. In accordance, the same organoids demonstrated decreased expression of the mesenchymal marker VIM and the proliferation marker Ki67 (Fig. 3). Interestingly, rapamycin treatment failed to promote differentiation of FAP2 cells which carry different types of APC mutation, as evidenced by low expression of the epithelial markers CDX2 and CK20, and a complete absence of the colon goblet cell marker MUC2. This poor differentiation of FAP2 cells even following rapamycin treatment was accompanied by their proliferation (Ki67), and the presence of mesenchymal cells (Vimentin-positive cells) similar to untreated FAP2 (Fig. 3). While rapamycin-treated FAP2 organoids exhibited more ‘complex’ structures, these structures lacked expression of the intestinal markers CDX2 and CK20, indicating random rather than directed differentiation.

**Fig. 4:**
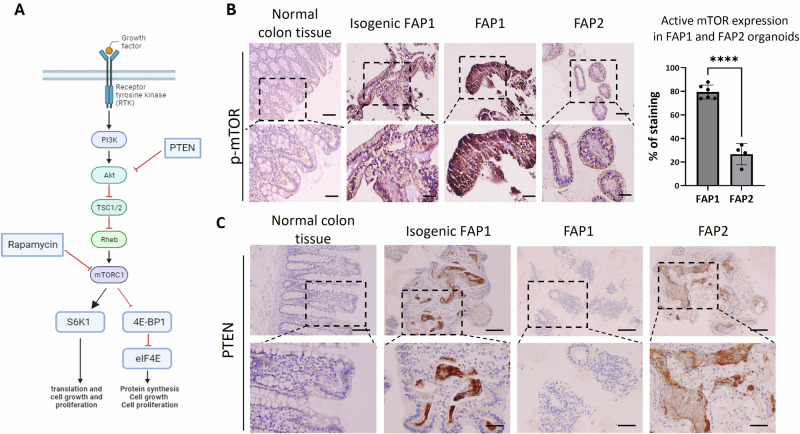
Expression of mTOR and its upstream suppressor PTEN, in colon organoids. **A** The schematic diagram for mTOR signaling pathway. In response to growth factors, PI3K is activated and phosphorylates AKT, which inactivates the tuberous sclerosis (TSC) tumor suppressor protein, the RAS homolog enriched in brain (Rheb) small G protein which activates mTOR. Activated mTOR phosphorylates S6K1 kinase which activates translation, cell growth, and proliferation. In parallel, phosphorylation and inactivation of 4E-BP1 activates eIF4E which leads to protein synthesis, cell growth, and proliferation**. B** Immunohistochemical staining of phospho-mTOR (p-mTOR) in normal colon tissue, and day 45 organoids derived from isogenic control FAP1, FAP1, and FAP2. Normal colon tissue served as a control for protein expression within the normal environment. This experiment was repeated twice, and representative pictures are shown. p-mTOR expression quantification utilized the open-source image analysis software Ilastik (version 1.4.1rc2), ****P < 0.0001, *t* test. **C** Immunohistochemical staining of the tumor suppressor protein PTEN in day 45 organoids derived from isogenic FAP1, FAP1, FAP2, and normal colon tissue served as a control. X10- Scale bars- 100 µm, X20- Scale bars- 200 µm.

To elucidate the mechanisms underlying rapamycin’s differential effect on FAP1 versus FAP2 differentiation into colon organoids, we investigated the expression of key genes within the mTOR pathway. We compared heterozygous APC-FAP1 organoids to both FAP1 isogenic control cells (genetically corrected FAP1) and human normal colon tissue (Fig. 4). Our findings revealed a significantly higher expression of the active phosphorylated form of mTOR (p-mTOR) in FAP1 organoids with the heterozygous APC mutation, compared to control groups (i.e. genetically corrected FAP1 and normal colon tissue), as well as to FAP2 cells, demonstrate a significant increase in the expression of active phosphorylated mTOR in FAP1 colon organoids compared to FAP2 (Fig. 4B). While no p-mTOR expression was detected in control normal colon tissue, corrected FAP1 organoids exhibited some level of expression, that is likely attributed to the presence of EGF in the differentiation medium, an mTOR pathway activator.

To unravel the underlying mechanism behind mTOR pathway overexpression in FAP1 organoids, we examined PTEN, a crucial tumor suppressor gene that negatively regulates mTORC1. Interestingly, PTEN was expressed in both genetically corrected FAP1 and FAP2 organoids but was undetectable only in FAP1 organoids. This absence of PTEN might explain the selective response of FAP1 to rapamycin treatment, which targets the mTOR pathway (Fig. 4C). Moreover, PTEN expression within FAP2 cysts (and not in FAP1), might contribute to the detrimental effect of rapamycin even on cyst formation. The distinct APC mutations in each cell line explain these observed differences; heterozygous APC mutations lead to varied downstream signaling, with mTOR activation being mutation specific. FAP1’s lack of PTEN, likely drives its high mTOR activity and sensitivity to rapamycin. In contrast, FAP2 expresses PTEN and does not show mTOR pathway hyperactivation. Consequently, rapamycin treatment did not improve its phenotype, suggesting that mTOR signaling is not a primary driver of cancer-related features in FAP2. These findings underscore the importance of understanding the molecular context and pathway dependencies of specific mutations when considering targeted therapies.

**Fig. 5:**
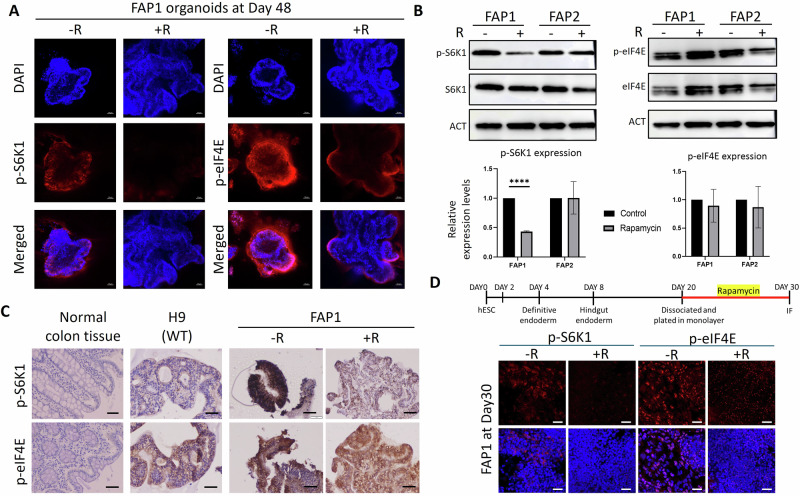
Rapamycin decreases the expression of the mTOR target proteins p-S6K1 and p-eIF4E, in FAP1 organoids. **A** Confocal immunofluorescence images of day 48 FAP1 colon organoids for p-S6K1 and p-eIF4E. This experiment was repeated twice, and 2-3 organoids were analyzed from each experiment. Representative images are presented. Scale bars- 50 µm. **B** Western blot of phosphorylated S6K1 and phosphorylated eIF4E for FAP1 and FAP2 organoids on day 48, in the presence/absence of rapamycin. The experiment was repeated twice, quantified by ImageJ, and normalized to the non-phosphorylated protein. ****P < 0.0001, *t* test. **C** Immunohistochemical analysis of p-S6K1 and p-eIF4E in day 48 colon organoids derived from a control hESC line (H9), and FAP1 with/without rapamycin. Normal colon tissue served as a positive control. Scale bar- 100 µm. Two independent biological experiments were performed, and representative data are shown. **D** Confocal immunofluorescence analysis of FAP1 cells differentiated in monolayer from day 20 to day 30, with and without rapamycin. Three independent biological experiments were performed. Scale bars- 50 µm.

To validate our hypothesis that the heterozygous APC mutation in FAP1 cells increased mTOR signaling, leading to impaired differentiation, which may be partially rescued by rapamycin treatment, we examined the expression levels of p-S6K1 and p-eIF4E, downstream effectors of the mTOR pathway. Immunofluorescence staining of whole organoids revealed the presence of both p-S6K1 and p-eIF4E within the cyst-like structures derived from FAP1 cells. Consistent with our hypothesis, rapamycin treatment significantly reduced the expression level of p-S6K1 and p-eIF4E (Fig. 5A). Western blot analysis corroborated these observations, revealing a greater than two-fold decrease in p-S6K1 protein levels in FAP1 cells treated with rapamycin compared to untreated controls (Fig. 5B). In contrast to p-S6K1, rapamycin treatment did not alter p-eIF4E protein levels as assessed by Western blot (Fig. 5B), but immunohistochemistry revealed a more balanced cellular distribution of p-eIF4E (Fig. 5C). Confocal microscopy of rapamycin-treated FAP1 cells revealed a decrease in active S6K1 and eIF4E even at these earlier stages, suggesting that rapamycin may exert its influence on differentiation from the beginning of the process (Fig. 5D). Our results indicate that while Western blot analysis showed no change in total eIF4E levels following rapamycin treatment (Fig. 5B), IHC analysis suggests a potential shift in the subcellular localization of active eIF4E (Fig. 5C). Specifically, IHC analysis reveals a change from a more localized, strong eIF4E expression primarily at the organoid edges to a more even distribution throughout all cell compartments after rapamycin treatment. This observation is further supported by the reduced expression of active/p-eIF4E seen in both 3D colon organoids (Fig. 5A) and monolayer cultures of differentiated colonic cells (Fig. 5D).

The indifference in the mTOR downstream proteins p-S6K1 & p-eIF4E between FAP1 and FAP2 can be explained by the activation of these downstream proteins by other signaling pathways rather than mTOR [29–31].

In contrast to FAP1 organoids, rapamycin treatment in FAP2 organoids did not induce any significant changes in the expression levels of either p-S6K1 or p-eIF4E compared to untreated control (Fig. 5B). Collectively, our findings suggest that rapamycin promotes the enhanced differentiation capacity of FAP1 organoids through its effect on p-S6K1.

**Fig. 6:**
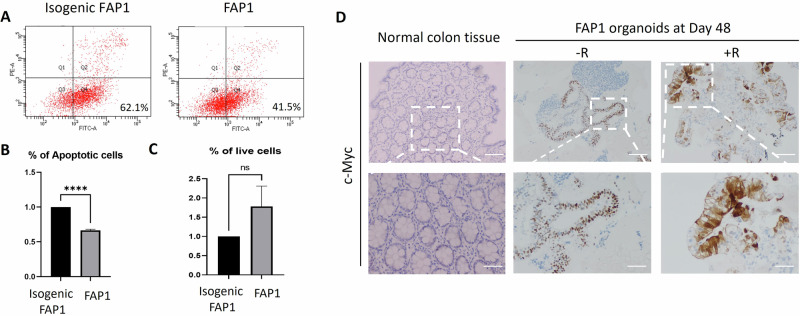
FAP1 differentiated cells inhibit apoptosis through the inactivation of cMyc. **A** FACS analysis plots of apoptotic cells, analyzed by Annexin-V (x-axis, FITC-A), and necrotic cells analyzed by PI (y-axis, PE-A), at day 20 of differentiation, using the MEBCYTO Apoptosis Kit (Annexin V-FITC Kit). A total of 10,000 cells of each line were analyzed and shown is a representative plot. **B**, **C** Quantification of FACS analysis for apoptosis (**B**) and live cells (**C**), of three independent experiments. ****P < 0.0005, *t* test. **D** Immunohistochemical analysis of c-Myc expression in day 45 FAP1 colon organoids with/without rapamycin (±R). Normal colon tissue served as a negative control. This experiment was repeated twice, with the analysis of 2–3 pathological sections.

Beyond its well-established function in promoting cell proliferation, mTOR can also exert anti-apoptotic effects when highly activated [32, 33]. Therefore, the viability, apoptotic, and necrosis of differentiated FAP1 cells were compared with those of their genetically corrected counterparts. Results indicate that differentiated FAP1 cells exhibit increased resistance to apoptosis compared to control cells, as demonstrated by a lower apoptotic cell count and a higher number of viable cells (Fig. 6A–C). These findings suggest that mTORC1 activation, as observed in cancerous FAP1 cells, may contribute to their enhanced resistance to cell death.

The anti-apoptotic effect of mTOR can be mediated by activating the translation of the oncogenic c-Myc, a multifunctional transcription factor primarily localized in the nucleus to drive cell proliferation [34–36]. Our results show that while normal control colon tissue doesn’t express c-Myc, this oncogene is expressed and active in the nucleus of FAP1 colon organoid cells. More important, rapamycin treatment of FAP1 organoids induced c-Myc translocation to the cytoplasm of the cells, out of its active site (Fig. 6D). These results further prove the effect of rapamycin on c-Myc trans-localization from the nucleus to the cytoplasm, to decrease its oncogenic effect.

**Fig. 7:**
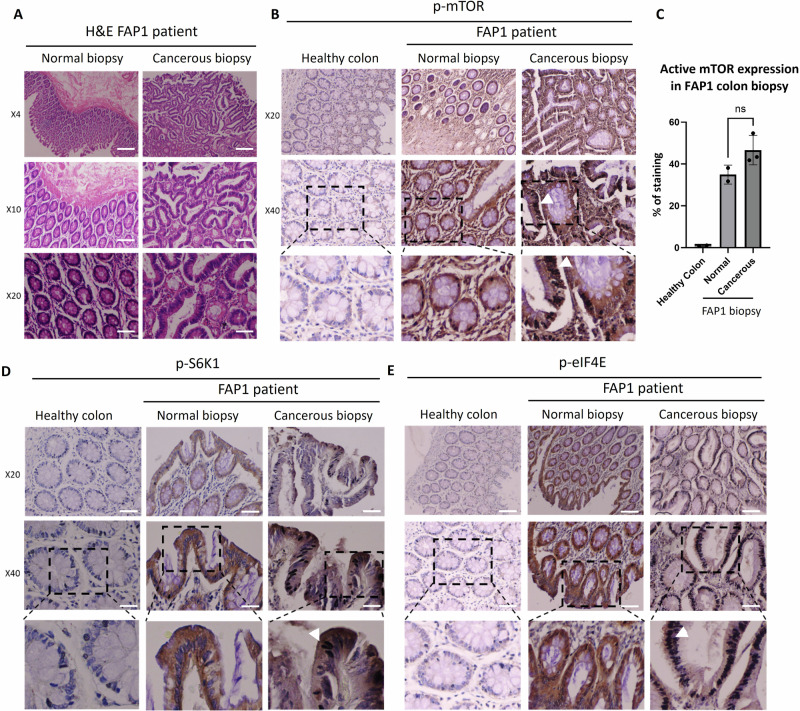
mTOR pathway genes expression in the colon of FAP1 patient. **A** H&E staining of normal and cancerous colon biopsies from FAP1 patient. **B** Representative immunohistochemical staining of p-mTOR **(C)** p-mTOR expression quantification utilized the open-source image analysis software Ilastik. **D**, **E** Representative immunohistochemical downstream protein p-eIF4E in normal and cancerous FAP1 patient biopsies. White arrows indicate regions of high mTOR pathway gene expression. Healthy colon tissue served as a control. X4 Scale bars- 500 µm, X10 Scale bars- 200 µm, X20 Scale bars- 100 µm, X40 Scale bars- 50 µm.

Encouraged by these in vitro results, we next sought to investigate mTOR pathway activation in the colon of FAP patients, to assess the translational relevance of our human in vitro organoid model findings to the human disease state. Histological analysis of H&E-stained colon biopsies revealed a well-organized villus-crypt architecture in the normal tissue obtained from the FAP1 patient, and loss of this organized structure in the cancerous biopsy from the same patient (Fig. 7A). Interestingly, p-mTOR levels were elevated not only in the cancerous FAP1 biopsies but also in the normal biopsy of the same FAP1 patient. In contrast, p-mTOR is nearly absent in the colon of healthy controls. Furthermore, cancerous FAP1 colon exhibited even higher p-mTOR expression, specifically in their elongated and enlarged nuclei (Fig. 7B). We highlight the unique opportunity afforded by access to colon samples from the same FAP1 patient who donated embryos for hESC derivation. This access, obtained with ethical approval and informed consent, enabled our novel genotype/phenotype correlation analysis, directly comparing in vitro organoid-SC model findings with in vivo colon tissue from the patient carrying the same mutation (Fig. 7C). Consistent with these findings, p-eIF4E and p-S6K1 were highly expressed in the elongated nuclei of cancerous FAP1 cells. An expression was also observed in the cytoplasm of the healthy FAP1 tissue and was absent in control biopsies from healthy individuals (Fig. 7D, E). These results indicate that the colon of FAP1 patient strongly activates the mTOR signaling, compared to the colon of healthy patient. These results emphasize the difference between the early stages of cancer development observed in the normal biopsy of FAP1 patient which carries the heterozygous APC mutation and the late stage apparent in the cancerous biopsy. Our results identify a potential therapeutic role for rapamycin in a specific subset of FAP1 patients characterized by a truncated APC and constitutively active mTOR signaling.

## Discussion

Activation of the mTOR signaling pathway is common in multiple human cancers such as breast [37], prostate [38], bone [39], lung [40], and colon. It is critical in promoting cell proliferation-related protein synthesis, lipid metabolism, mRNA translation and thus mediates cancer cell proliferation [41, 42]. Prior studies have implicated hyperactive mTOR signaling as a key driver of tumorigenic transformation in CRC via its potent activation of downstream effector proteins, p70S6K and 4EBP1 [41, 43].

FAP1-hESCs harboring a nonsense mutation leading to premature protein truncation at arginine 332 demonstrated enhanced differentiation into mature complex colon organoids upon treatment with the mTOR inhibitor rapamycin. This rapamycin-mediated rescue of cellular phenotype was specific to FAP1 stem cells characterized by a truncated APC mutation and hyperactive mTOR signaling and was absent in FAP2 stem cells carrying a different APC mutation without mTOR pathway activation. mTOR inhibitors have emerged as promising therapeutic candidates for restoring apoptotic sensitivity and suppressing tumor growth, particularly in CRC [17, 44]. Here, we suggest using rapamycin as a potential strategy to delay or prevent tumorigenic transformation in colon epithelial cells exhibiting high levels of mTOR activity. The effective rapamycin concentration for mTOR suppression and substrate inhibition varies significantly across different cell lines [45]. Consistent with the aforementioned variability in rapamycin efficacy, we observed that a nanomolar dose effectively inhibited S6K1 phosphorylation, a direct substrate of mTORC1. Conversely, although Western blot analysis showed no significant change in eIF4E phosphorylation (a downstream target of 4E-BP1), immunofluorescence suggested potential alterations in its subcellular localization. These results support the notion that nanomolar concentrations of rapamycin can selectively inhibit certain mTORC1 substrates, like S6K1, while not fully suppressing signaling pathways involving 4E-BP1.

Rapamycin, a well-established pharmacological inhibitor of mTOR signaling, has been FDA-approved as an immunosuppressant since 1999 [46, 47]. Several rapamycin analogs, including temsirolimus (approved for advanced renal cell cancer)[48] and sirolimus (currently in phase II and III clinical trials), are being investigated as potential cancer treatments [49]. Rapamycin has been shown to significantly extend lifespan in APC^+/−^ mice model of FAP, by reducing colon neoplasia [27]. Animal models serve as a valuable platform for investigating CRC progression and evaluating therapeutic interventions within the complex, whole-organism context [50, 51]. Conversely, human cell-based systems provide mechanistic insights into disease pathogenesis and enable a more direct assessment of potential treatment strategies. The genetic background influences tumor microenvironment, cancer progression, and drug response differently in mice compared to humans. In addition, mice typically exhibit a lower incidence of polyps, predominantly located in the small intestine, compared to humans who develop a higher number of polyps primarily in the colon and rectum [52]. Therefore, elucidating the mechanisms underlying disease onset and progression in a human context is crucial.

Here, using our human in vitro model, we show that FAP2 organoids, lacking prominent mTOR expression, exhibited no significant response to rapamycin. The presence of active PTEN, a tumor suppressor known to negatively regulate mTOR, in FAP2 organoids may contribute to their unresponsiveness to rapamycin treatment. These findings indicate that mTOR pathway activation is not a universal consequence of APC germline mutations, at least during the early stages of tumorigenesis as modeled by our colon organoid system. The successful rescue of the FAP1-colon organoid phenotype by rapamycin correlates with the absence of PTEN expression in these cells. It is noteworthy that PTEN silencing has been reported in approximately 6% of advanced-stage cancer patients [53]. Our findings demonstrate the importance of PTEN status in determining response to rapamycin, highlighting the potential for personalized treatment strategies based on PTEN expression.

The mTOR signaling pathway orchestrates a wide range of metabolic processes by activating transcription factors, including the oncogenic c-Myc [34, 35, 54]. c-Myc expression is tightly controlled in normal tissues but is aberrantly upregulated in approximately 70% of human cancers [55]. mTORC1 activation stimulates c-Myc mRNA translation, leading to increased c-Myc protein abundance. This increase enhances cellular metabolic activity and growth while concurrently suppressing apoptotic signals, thereby promoting cell survival in conditions that would normally induce apoptosis [17, 56]. Moreover, rapamycin plays a critical role in c-Myc mRNA translation by inhibiting cap-dependent translational initiation and the formation of the ribosomal 48S initiation complex [57]. Hyperactive mTORC1 signaling strongly correlates with increased nuclear c-Myc expression in an APC-KRAS mutant mouse model. Moreover, rapamycin treatment has been shown to effectively reduce nuclear c-Myc levels [58]. Similarly, our FAP1 organoids exhibited nuclear c-Myc localization, which was redirected to the cytoplasm upon rapamycin treatment. Notably, c-Myc is transported from the nucleus to the cytoplasm for proteasomal degradation [59].

As we have previously reported, FAP1 patients exhibit severe clinical manifestations of the disease, including an increased polyp burden [28]. To further prove the therapeutic potential of rapamycin for FAP patients harboring APC mutations that disrupt mTOR signaling, we examined mTOR pathway activation in colon biopsies from FAP1 patient. Interestingly we found elevated mTOR protein levels and increased phosphorylation of its downstream effectors S6K1 and eIF4E, in both the cancerous and non-cancerous colon biopsies from FAP1 patients. These findings suggest that mTOR pathway activation is an early event in FAP1 pathogenesis, preceding the development of adenomas and carcinomas. Furthermore, our data indicate that mTOR expression and localization evolve over time, with increased nuclear localization accompanying disease progression. Our in vitro findings using human colon-derived cells provide a strong preclinical foundation for the establishment of clinical trials aimed at delaying or preventing the adenoma-carcinoma transition in FAP1 patients carrying similar APC mutations.

## Data Availability

This published article and its supplementary information files include all data generated or analyzed during this study.
